# Rippling life on a dormant planet: hibernation of ribosomes, RNA polymerases, and other essential enzymes

**DOI:** 10.3389/fmicb.2024.1386179

**Published:** 2024-05-06

**Authors:** Karla Helena-Bueno, Lewis I. Chan, Sergey V. Melnikov

**Affiliations:** Biosciences Institute, Newcastle University, Newcastle upon Tyne, United Kingdom

**Keywords:** hibernation, stress, ribosome, Rubisco, RNA polymerase, dormancy

## Abstract

Throughout the tree of life, cells and organisms enter states of dormancy or hibernation as a key feature of their biology: from a bacterium arresting its growth in response to starvation, to a plant seed anticipating placement in fertile ground, to a human oocyte poised for fertilization to create a new life. Recent research shows that when cells hibernate, many of their essential enzymes hibernate too: they disengage from their substrates and associate with a specialized group of proteins known as hibernation factors. Here, we summarize how hibernation factors protect essential cellular enzymes from undesired activity or irreparable damage in hibernating cells. We show how molecular hibernation, once viewed as rare and exclusive to certain molecules like ribosomes, is in fact a widespread property of biological molecules that is required for the sustained persistence of life on Earth.

## Just like bears and arctic squirrels, certain cellular enzymes can hibernate too

Do you know that we live on a dormant planet? It is currently estimated that at least 60% of the global microbial biomass exists in various forms of dormancy (Blagodatskaya and Kuzyakov, 2013; Rittershaus et al., 2013). Some bacteria can use dormancy to remain viable for astonishing stretches of time, in some cases exceeding 250 million years (Vreeland et al., 2000; Morono et al., 2020). Furthermore, dormancy is not limited to the realm of microbes. For a multitude of multicellular organisms, including bears, arctic squirrels, raccoons, snakes, snails, spiders, and many others, hibernation serves as an integral stage in the normal life cycle (Nedergaard and Cannon, 1990; Lee, 2008; Mohr et al., 2020). Organisms that do not hibernate as a whole can induce dormancy in some parts of their bodies. For instance, in human bodies, oocytes can remain dormant for over 30 years, exhibiting no overt signs of life while being fully capable of initiating a new life in response to fertilization. Therefore, for many organisms the most prevalent form of life is lack of any activity.

**Figure 1:**
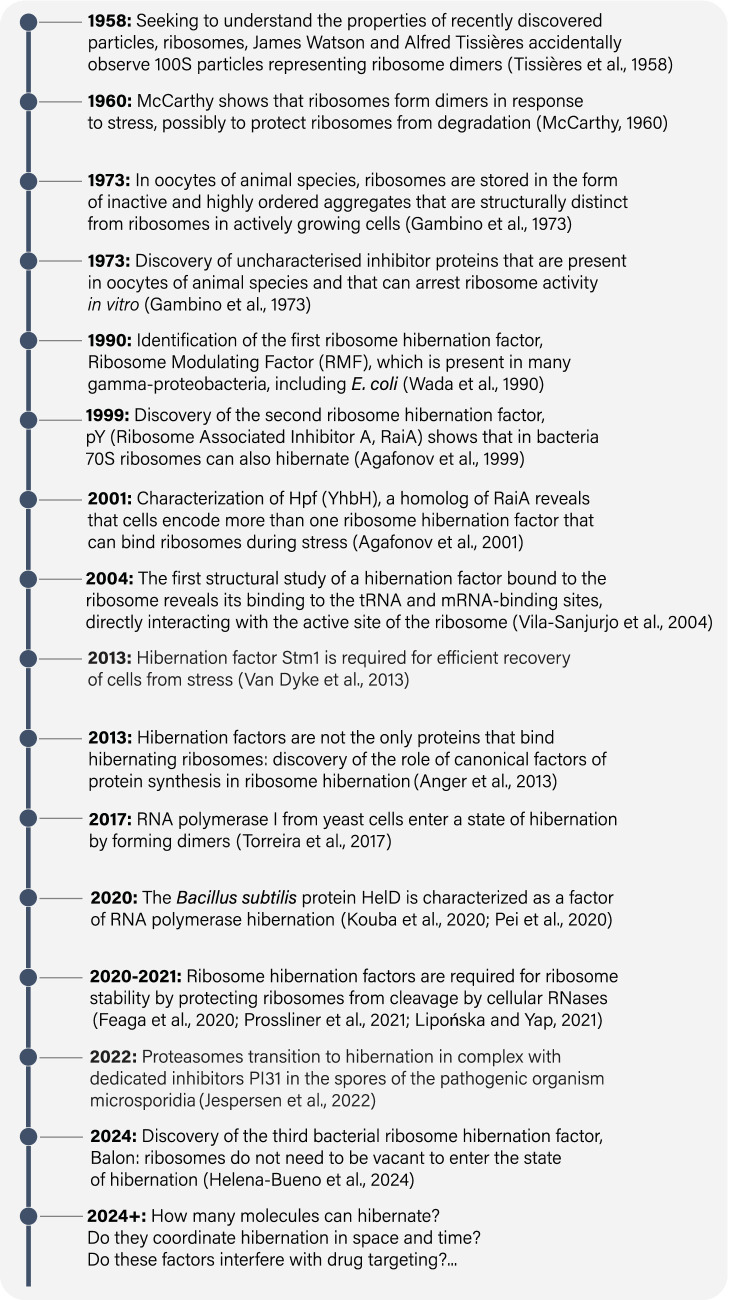
A timeline of some major milestones in studies of hibernating molecules.

Decades ago, clues began to emerge from early studies of central biomolecules that dormant cells contain dormant molecular machines (Figure 1). However, many of these clues were collected independently, by scientists who had little awareness of studies by others while being focused on their preferred biological assembly—whether it be the ribosome, RNA polymerase, proteasome, ATP synthetase, or others (McCarthy, 1960; Pullman and Monroy, 1963; Hille, 1974; Patterson et al., 1984; Gutteridge et al., 1986; Chu-Ping et al., 1992; Fernández-Tornero, 2018). Nevertheless, when combined, research spanning from the late 1950s to the present day shows compelling evidence of the existence of a common molecular mechanism that allows organisms to survive in a state of dormancy. We refer to this mechanism as “hibernation of biological molecules.” This concept refers to a self-preservation strategy that is conserved from the simplest bacteria to humans, which consists in the production of a special class of proteins, known as hibernation factors that either inhibit or protect essential biological molecules from degradation in response to starvation and stress. And it is only through the presence of these hibernating proteins that organisms can endure extended periods of decreased metabolic activity without digesting and degrading their most essential molecular structures that are indispensable for a cell to be alive.

Since our planet is teeming with dormant life, we anticipate the studies of hibernating biological molecules to bring a lot of value to the clinic and the commercial world. By elucidating how hibernating molecules enable pathogenic bacteria to withstand stress and assaults from antibiotics, we hope to learn how to effectively combat chronic infections by subverting pathogens’ mechanisms of molecular hibernation—something that has already been accomplished to some degree (Song et al., 2019). By studying how this process protects cells and tissues, including agricultural seeds, transplant tissues, or human oocytes, we hope to learn how to enhance biological dormancy processes to slow aging and preserve valuable biological specimens in a safer and longer-lasting form. Underlying all these practical applications, the study of molecular hibernation promises to illuminate the underpinnings of a fundamental mechanism sustaining life on Earth. With the rapid advances in imaging and other tools to study hibernation, we anticipate uncovering an intricate process that prepares a living cell for dormancy—allowing biological molecules to perform their most common activity, which paradoxically involves no activity at all.

## Hibernating ribosomes were discovered by chance, as a by-product of ribosome isolation

In 1958, James Watson worked with Alfred Tissières to study ribosomal particles, which had been recently discovered by George Palade (Tissières et al., 1959). To characterize the chemical properties of ribosomes and the conditions contributing to their stability *in vitro*, they isolated ribosomes from actively growing *Escherichia coli* cultures. However, their experimental procedure involved placing *E. coli* culture on ice and pelleting the cells before isolating the ribosomes. As a result, *E. coli* cells were inadvertently exposed to cold shock, hypoxia, and nutrient depletion. Under these conditions, Tissières and Watson observed 100S particles in addition to individual 70S ribosomes. They correctly concluded that 100S particles represent ribosome dimers, but they were unaware of their biological significance (Tissières et al., 1959). Thus, unknowingly, Tissières and Watson became perhaps the first people on Earth to observe hibernating molecules. Their accidental observation initiated a long-standing tradition of discovering hibernating molecules by chance, as a byproduct of their isolation from cells or tissues that were placed on ice and centrifuged in nutrient-free buffers (Yusupov and Spirin, 1988; Ben-Shem et al., 2011; Anger et al., 2013; Brown et al., 2018; Barandun et al., 2019; Ehrenbolger et al., 2020; Nicholson et al., 2022).

Two years later, Brian McCarthy showed that *E. coli* 100S ribosomes appear only during stationary phase or nutrient depletion, when *E. coli* cells reduce the rate of protein synthesis (McCarthy, 1960). Upon nutrient replenishment, cells promptly resumed protein synthesis, and the presence of 100S ribosomes was no longer observed. These experiments put forth the idea that environmental stress might govern the formation of 100S ribosomes, which were only detectable during periods of protein synthesis arrest: laying an early foundation for our current understanding of ribosome hibernation. Additionally, McCarthy was the first to hypothesize that cells may possess the ability to “switch off” their ribosomes in response to stress (McCarthy, 1960).

## Ribosomes in metabolically inactive bacteria associate with hibernation factors

While the mechanism behind the formation of *E. coli* 100S ribosomes would remain a mystery for over 3 decades, important strides were made during that time in other experimental systems, including unfertilized eggs of animal species. By that time, it was well established that oocytes can endure dormancy for several decades while remaining capable of rapid reactivation upon fertilization. Seeking to understand this remarkable capacity for self-preservation, early studies in the 1970s detected “factors” of unknown molecular identity that bind to ribosomes in unfertilized eggs and inhibit protein synthesis *in vitro* (Gambino et al., 1973; Hille, 1974; Huang and Warner, 1974). Thus, as early as 1973, it became clear that ribosomes can associate with unidentified factors that seem to inhibit protein synthesis in dormant, unfertilized eggs.

The first ribosome hibernation factor was identified in 1990 by Wada et al. (1990). Using two-dimensional protein gels to characterize the composition of *E. coli* ribosomes under different growth conditions, they discovered an additional protein that binds to ribosomes from the 100S fraction of stationary *E. coli* cells in a stoichiometric manner. They named this tiny protein, comprising just 55 amino acids, ribosome-modulating factor (RMF). They showed that RMF gene is transcriptionally activated in stationary phase cultures and is necessary for the formation of 100S ribosomes in *E. coli*. Based on these findings, Wada and colleagues were the first to propose that RMF triggers the formation of 100S ribosomes as a strategy to store and protect ribosomes from nucleolytic degradation. Therefore, the research conducted by Wada’s group established that, in metabolically inactive cells, nearly all (or all) ribosomes associate with a dedicated hibernation factor.

**Figure 2:**
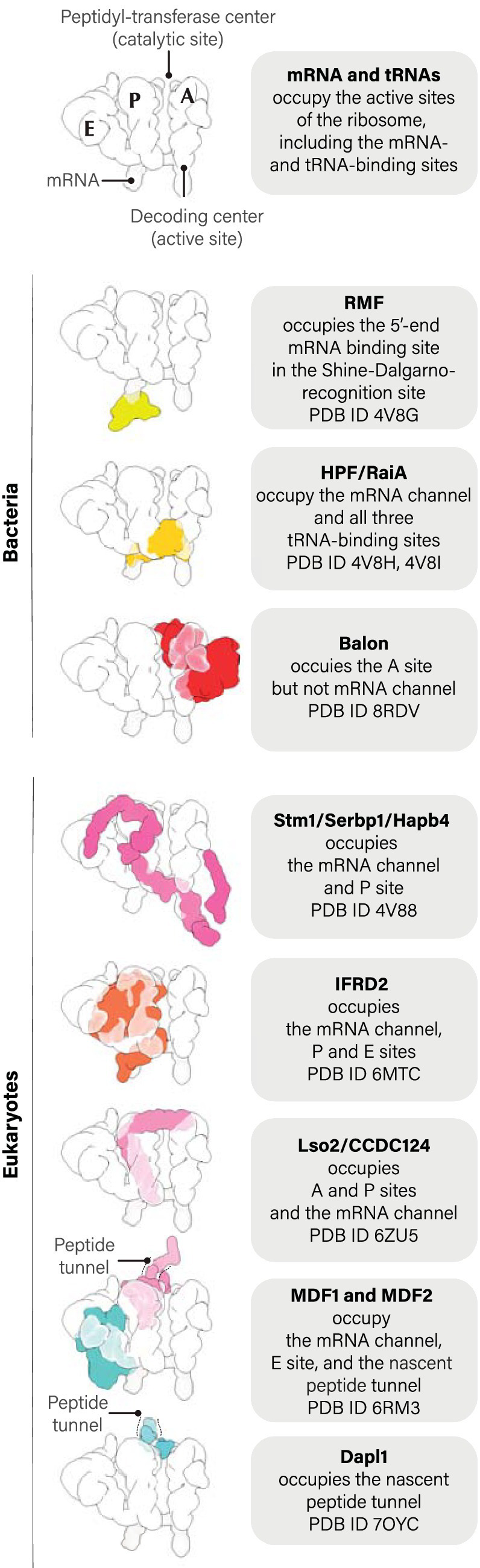
Currently known ribosome hibernation factors and their ribosomal binding sites.

Shortly after the identification of RMF, two additional ribosome hibernation factors, HPF and RaiA, were identified in *E. coli* (Figure 1; Table 1). HPF and RaiA share the same fold and likely originated through gene duplication to fulfill redundant roles in ribosome hibernation (Agafonov et al., 1999, 2001; Maki et al., 2000). In 2004, RaiA became the first ribosome hibernation factor to be structurally characterized while bound to ribosomes. The pioneering study by Jamie Cate and colleagues showed that RaiA occupies the active sites of the small ribosomal subunit, including the tRNA-binding sites and mRNA-binding channel (Figure 2; Vila-Sanjurjo et al., 2004). This structural study revealed a common characteristic that was later identified in virtually all known ribosome hibernation factors: not only do these factors bind to ribosomes in metabolically inactive cells, but also they occupy the active centers of ribosomes, making these centers inaccessible for other biological molecules.

**Table 1:** How many names is enough for a single protein family?

Protein name	Representative organism	Gene ID	Name origin	References
pY	*Thermus thermophilus/Escherichia coli*	TTHA0270/yfiA	Because this protein was discovered by Marat Yusupov and colleagues during their studies of *Thermus thermophilus* ribosomes, it was named “Protein Yusupov” or pY. Later, homologous proteins were found in other bacteria, where they are sometimes referred to as pY.	Yusupov and Spirin (1988)
RaiA	*Escherichia coli*	yfiA	Early studies have established the inhibitory impact of this protein on *in vitro* protein synthesis. Hence, it was also named “Ribosome associated inhibitor A.”	Agafonov et al. (2001)
YfiA	*Escherichia coli*	yfiA	Some refer to this protein using its gene name, using the uniform nomenclature proposed and developed by Demerec and colleagues.	Agafonov et al. (1999)
Long HPF	*Escherichia coli*	yfiA	Proteins RaiA and HPF from *E. coli* belong to the same protein family and have a similar structure, except for the C-terminal protein extension in RaiA that is absent in HPF. To distinguish between HPF and RaiA and their homologs in other species, many prefer to use the term “short” and “long” HPF to indicate that the short one is similar to *E. coli* protein HPF (known to cause the formation of ribosome dimers with protein RMF), as opposed to protein RaiA (that makes ribosomes hibernate in their monomeric state).	Maki and Yoshida (2021)
YhbH	*Escherichia coli*	yhbH/hpf	Named after its gene.	Maki et al. (2000)
HPF	*Escherichia coli*	yhbH/hpf	Due to its ability to bind to ribosomes in metabolically inactive cells and induce formation of ribosome dimers (where HPF acts in cooperation with RMF), this protein was named as Hibernation Promoting Factor.	Polikanov et al. (2012)
Short HPF	*Escherichia coli*	yhbH/hpf	Same origin as the “long HPF.”	Sato et al. (2009)
Msmeg_3935	*Mycobacterium smegmatis*	Msmeg_3935	In the dystopian novel “Brave New World” by Aldous Huxley characters are named by numbers instead of names. Similarly, in *M. smegmatis* studies, proteins are typically identified by their gene names, reflecting their designated number in mycobacterial genomes. For instance, “Msmeg_3935” denotes gene number 3935 in *M. smegmatis*.	Trauner et al. (2012)
Ribosomal protein S30AE	*Mycobacterium smegmatis*	Msmeg_3935	Some databases and research articles use the term “ribosomal protein S30AE” to denote this hibernation factor. This naming is based on the structural resemblance of HPF/RaiA and the protein to archaeao-eukaryotic ribosomal protein S30, which likely stems from a shared evolutionary origin between S30 and HPF/RaiA.	Trauner et al. (2012)
mpY	*Mycobacterium smegmatis*	Msmeg_3935	Using the original name “protein Y,” some studies call the mycobacterial homologs of this protein “mpY” to add some mycobacterial flavor.	Li et al. (2018)
RafH	*Mycobacterium smegmatis*	Msmeg_3935	One established role of hibernation factors in mycobacteria is to aid their survival under hypoxic conditions by protecting their ribosomes. Therefore, a study proposed to name this protein “ribosome-associated factor under hypoxia” or RafH.	Kumar et al. (2024)
Psrp1	*Spinacia oleracea*	PSRP1	Initially, this HPF/RaiA-type protein was mistakenly designated as a plant-specific ribosomal protein 1 (Psrp1). However, it was later discovered to be a chloroplast-specific ribosome hibernation factor. The name, however, is still in use not only for its homologs from chloroplasts in other species but also for those found in bacteria, especially photosynthetic cyanobacteria.	Sharma et al. (2010)
LrtA	*Arabidopsis thaliana/*Cyanobacteria	lrtA	Because the activity of this hibernation factor, belonging to the HPF/RaiA type, is regulated by light in chloroplasts of plants and in cyanobacteria, it was named Light-repressed protein A.	Contreras et al. (2018)
Rv0079	*Mycobacterium tuberculosis*	Rv0079	Similarly to Msmeg_3935 from *M. smegmatis*, HPF/RaiA is named in *M. tuberculosis* after its gene.	Kumar et al. (2012)

In the story of the Tower of Babel, God decides to punish arrogant people by inventing new languages to prevent them from understanding each other. It seems that a similar issue exists in the field of ribosome hibernation, where homologs of the same protein have different names in different organisms or even in the same organism (with Mycobacterium smegmatis being the prime example). To help our readers, we have created a catalog of the most common names of proteins from the HPF/RaiA family. We show that this craving for pseudonyms arises from independent discoveries of the same protein in different species or is driven by the fact that some species encode more than one isoform of HPF/RaiA factors, creating a need to discriminate between them.

## Ribosome hibernation factors are not limited to bacteria but also exist in eukaryotes

Following the discovery of ribosome hibernation factors in bacteria, functionally similar proteins were soon identified in eukaryotic species. The first of these factors, Stm1, was identified in 2011 as a protein that binds to essentially all cellular ribosomes in yeast *S. cerevisiae* in response to sudden glucose starvation (Ben-Shem et al., 2011). Later, the Stm1 homolog, Serbp1, was found to bind to ribosomes in cold-shocked cells of humans and *Drosophila melanogaster* (Anger et al., 2013). Subsequent studies revealed five more families of ribosome hibernation factors. These included (i) proteins Lso2/CCDC124 in humans, yeasts and parasitic fungi microsporidia (Wang et al., 2018; Ehrenbolger et al., 2020; Wells et al., 2020), (ii) proteins IFRD1/IFRD2 in rabbit reticulate extracts and *Drosophila* cells (Brown et al., 2018; Hopes et al., 2022), (iii, iv) proteins MDF1 and MDF2 in metabolically inactive spores of fungal parasites microsporidia (Barandun et al., 2019), and (v) protein Dap1b in frogs/Dapl1 in xenopus that participates in ribosome hibernation in oocytes of frogs or zebrafish (Leesch et al., 2023).

All these ribosome hibernation factors in eukaryotes were shown to act in essentially the same manner as bacterial hibernation factors—in terms of their ability to occupy the functional centers of all or most ribosomes in metabolically inactive or stressed cells (Figure 2). However, strikingly, none of the ribosome hibernation factors of eukaryotes bore resemblance to those of bacteria, indicating an independent evolutionary origin of ribosome hibernation factors in the two domains of life.

Combined with the studies of bacterial hibernation factors, these findings have taught us two important lessons. Firstly, it has become evident that the majority of characterized species harbor more than one family of hibernation factors. The best-studied organism *E. coli* stands out as the current leader in this regard, harboring at least four families of ribosome hibernation factors, including proteins RMF, HPF/RaiA, and putative hibernation factors Sra (Wada et al., 1990) and YqjD/ElaB/YgaM (Yoshida et al., 2012). The reasons underlying the necessity for multiple families of these proteins remain unclear. It also remains unclear whether each family of factors exhibits a preference for specific environments or stressors.

The second lesson learned is that, despite the essential role of ribosome hibernation in cell survival, these proteins are not universally conserved across the evolutionary tree. Instead, bacteria and eukaryotes possess distinct sets of ribosome hibernation factors—including Stm1/Serbp1 (Ben-Shem et al., 2011), Lso2/CCDC124 (Wang et al., 2018), IFRD1/IFRD2 (Brown et al., 2018), MDF1 (Barandun et al., 2019), MDF2 (Barandun et al., 2019), and Dap1b (Leesch et al., 2023) in eukaryotes, and HPF/RaiA (Agafonov et al., 1999, 2001; Maki et al., 2000), RMF (Wada et al., 1990), and Balon (Helena-Bueno et al., 2024) in bacteria.

It remains unclear how conserved each family of ribosome hibernation factors is across species: whether they are broadly conserved across bacteria or eukaryotes, or if certain lineages of bacteria and eukaryotes encode their own sets of hibernation factors. One difficulty in addressing this question lies in the much higher rate of sequence evolution for these proteins compared to other ribosome-binding proteins in a cell. This heightened rate of evolution makes it challenging to employ traditional approaches of homology search, such as simple Blast or even Markov Models-based search of homology, for the identification of their protein homologs across species (Helena-Bueno et al., 2024). However, the characteristic domain specificity of hibernation factors suggests that they emerged shortly after the split of bacteria and archaea-eukaryotes. If our current dating of this evolutionary event is accurate, then living cells have been enjoying ribosome hibernation for approximately 3.5 billion years.

## Hibernation factors support long-term survival by preventing ribosome degradation by nucleases

The discovery of ribosome hibernation factors made it possible to assess the physiological impacts of ribosome hibernation on cellular dormancy, metabolism, and survival. Studies have shown that the deletion of genes encoding RaiA/HPF and RMF leads to accelerated rRNA decay in metabolically inactive cells of *E. coli*, *Staphylococcus aureus*, and *Mycobacterium smegmatis* (Trauner et al., 2012; Lipońska and Yap, 2021; Prossliner et al., 2021). Furthermore, the absence of hibernation factors in stationary phase bacterial cultures was shown to cause ribosomal dissociation into individual subunits, followed by nucleolytic degradation of ribosomal subunits by the RNA-degrading enzyme RNase R (Lipońska and Yap, 2021). Remarkably, these ribosomes tend to accumulate rRNA nicks precisely at the sites where hibernation factors bind, leading to the conclusion that the primary role of ribosome hibernation factors is to safeguard the vulnerable active centers of the ribosome against cellular nucleases (Prossliner et al., 2021). The protective role of hibernation factors in maintaining ribosome integrity (as opposed to ribosome inhibition) has also been shown in resting *Bacillus subtilis* cells where lack of HPF results in a significant loss of ribosomal proteins uS2 and uS3 during stationary phase (Feaga et al., 2020). This effect is likely attributed to the degradation of the small ribosomal subunit by RNAse R (Dimitrova-Paternoga et al., 2024).

When dormant cells are transferred back to the normal growth environment, ribosomes quickly exit hibernation. Although the exact mechanism of this process is yet to be determined, studies of the hibernation factors HPF/RaiA, RMF, and Stm1 have shown that ribosomes may exit hibernation with the assistance of ribosome-recycling factors, including proteins RRF and HflX in bacteria (Basu and Yap, 2017; Basu et al., 2020), and Pelota/Dom34 and Rli1/Hbs1 in eukaryotes (Van Den Elzen et al., 2014). These factors split ribosomes into individual subunits and appear to release hibernation factors from the ribosomes, enabling them to engage in protein synthesis.

Shortly after the identification of hibernation factors in both bacteria and eukaryotes, it became evident that they impact the ability of cells to survive stress. Genetic knockouts of RMF in *E. coli* not only accelerate rRNA decay but cause a 100-fold decline in cellular survival rate after a 5-day toxic exposure to acid (El-Sharoud and Niven, 2007). The survival of RMF-deficient cells largely depends on the time that cells spend in stationary phase or under starvation. While RMF was dispensable for a relatively short-term stress of up to 4 days, the impact of RMF on the ability of cells to recover increased dramatically with longer periods of stress. A similar, time-dependent impact was observed in other model organisms, including Stm1(Serbp1)-deficient and Lso2-deficient yeasts (Van Dyke et al., 2013; Wang et al., 2018) and HPF/RaiA-depleted *E. coli* (Prossliner et al., 2021), *S. aureus* (Lipońska and Yap, 2021), and *M. smegmatis* (Trauner et al., 2012). Collectively, these data revealed that the fitness cost of hibernation factors depends on the duration of metabolically inactive states. The longer cells remain metabolically inactive in the absence of hibernation factors, the less likely they are to recover. Given that many cells can remain dormant for several years, hibernation factors are therefore a matter of life or death during such states of long-term dormancy.

Importantly, a growing body of evidence suggests that, aside from their role in long-term cell survival, hibernation factors may participate in short-term cellular responses. These responses include transient exposure to oxidants, zinc depletion, osmotic shock, cold shock, as well as gradual fluctuations of nutrients that are sensed by mTOR signaling or eEF2K signaling pathways (Li et al., 2018; Smith et al., 2021; Saba et al., 2023; Shetty et al., 2023). For example, eEF2K signaling appears to control ribosome availability in mammalian neurons by triggering transient hibernation of a subset of cellular ribosomes and inducing a reversible deposition of excessive amounts of ribosomes into large macromolecular condensates known as p-bodies (Smith et al., 2021).

## Hibernation factors like company: some factors of protein synthesis participate in hibernation too

**Figure 3:**
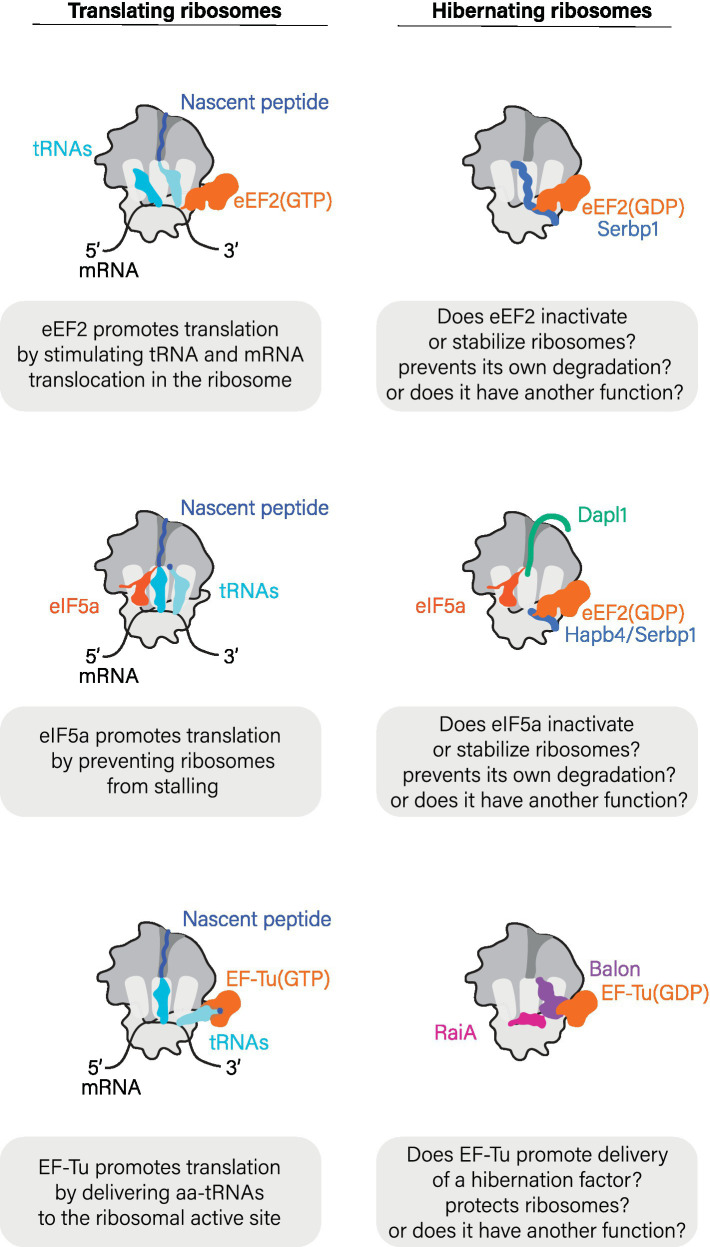
Hibernation factors frequently bind ribosomes in cooperation with factors of protein synthesis.

While foundational work suggested that ribosomes hibernate solely in association with hibernation factors, more recent research has shown that, for many biological species, this view is incomplete. Ribosomes isolated from ice-cold human blood samples and *Drosophila* eggs were shown to associate not only with the hibernation factor Serbp1 but with two additional molecules, the translation elongation factor eEF2 and deacylated tRNA (Figure 3) (Anger et al., 2013; Leesch et al., 2023). Similarly, ribosomes isolated from oocytes of frogs and zebrafish associate not only with the hibernation factors Serbp1(Hapb4) and Dapl1 in xenopus (frog) and dap1b in zebrafish, but also with eEF2 and the translation factor eIF5a (Leesch et al., 2023). In bacteria, the elongation factor EF-Tu has been shown to bind hibernating ribosomes in metabolically inactive *B. subtilis* cells during spore formation (Pereira et al., 2015), and in the γ-proteobacterium *Psychrobacter urativorans* where stress conditions trigger EF-Tu binding in addition to the hibernation factors HPF/RaiA and Balon (Helena-Bueno et al., 2024). The common thread connecting all these non-hibernation factors that bind dormant ribosomes is the fact that they are all accessory factors in normal protein synthesis, making their binding to hibernating ribosomes highly unexpected (Figure 3).

While the biological role(s) of these protein synthesis factors in ribosome hibernation remains unclear, a few lessons can be derived from structural studies that show the location of these factors in hibernating ribosomes. Just as during the normal cycle of protein synthesis, proteins eEF2 and EF-Tu bind to the sarcin-ricin loop and the L7/12-stalk (or P-stalk in eukaryotes) of hibernating ribosomes, whereas tRNA molecules associate with the ribosomal L1-stalk. It is therefore possible that these proteins may help to shield additional vulnerable active sites of hibernating ribosomes from undesired activity or damage. In light of these findings, the prevailing narrative has gradually shifted from viewing idle ribosomes as vacant to appreciating that idle ribosomes can bind a wide array of cellular proteins to actively enter an assisted state of hibernation.

## Social life of hibernating ribosomes

The large size of ribosomal particles, which enabled their early discovery by George Palade in 1955, has also enabled their observation through transmission microscopy in dormant cells. Among the earliest snapshots of this kind were the ones obtained in 1972 for oocytes and follicular cells of lizards *Lacerta sicula* (Taddei, 1972). These snapshots revealed that, during the periods of winter rest, the ribosomes of these lizards aggregate into so called “ribosome bodies.” Each of these bodies is made of crystalline sheets comprising thousands of ribosome tetramers that aggregate into a periodic pattern. During spring, these ribosome bodies are disassembled into individual ribosomes, so that ribosome bodies are completely absent in summer. Similar patterns were also observed in oocytes from mice (Burkholder et al., 1971) and an ascidian *Ciona intestinalis* (Kessel, 1966), as well as in cold-shocked chick embryos (Morimoto et al., 1972). There, ribosomes were found to be orderly packed on the inner side of their cell membranes, further illustrating that ribosomes can produce complex and periodical arrangements *in vivo*. In her Nobel Prize lecture, Ada Yonath, a pioneer in ribosome structural studies, revealed that early imaging studies of ribosomes through transmission electron microscopy served as a major motivation for her to attempt to crystallize ribosomes: the fact that ribosomes could form crystal-like aggregates in a cell suggested the possibility of their crystallization in a test tube (Yonath, 2010).

Following the initial observation of ribosome aggregates *in vivo*, these studies were largely forgotten and rarely discussed in the literature. However, with the advent of cryo-electron tomography, interest in this supramolecular organization of hibernating ribosomes has been reignited, bringing this intriguing ribosome behavior back into the spotlight of ribosome biology. Specifically, in the last year, cytosolic ribosomes of yeasts cell have been shown to associate with the outer membrane of mitochondria during glucose starvation (Gemin et al., 2023). Similarly, in metabolically inactive spores of parasitic fungi microsporidia, ribosomes were shown to assemble into helical sheets comprising dozens or hundreds of ribosomes per sheet (Sharma et al., 2024). Currently, the biological significance and mechanisms of these assemblies of hibernating ribosomes remain unclear. However, it is clear that hibernating ribosomes not only associate with special hibernation proteins but also undergo changes oligomeric status and intracellular localization, where they tend to associate with membranes and with each other.

## The economics of ribosome hibernation

How is the stoichiometry between ribosomes and hibernation factors regulated in metabolically active cells vs. dormant cells? Through quantifying ribosome levels in *E. coli* growing in different media, scientists from the so-called “Copenhagen School” in the early 1970s have found that in rich media, *E. coli* has a division time of 24 min and contains approximately 72,000 ribosomes per cell (Burton, 1998). In minimal media, the same strain had a division time of 100 min and contained only 6,800 ribosomes per cell. However, comparison of these cells revealed that more rapidly dividing *E. coli* tended to be much larger in size. As a result, the concentration of ribosomes within the cytoplasm of actively growing *E. coli* remains largely invariant, constituting nearly one-third of the dry cellular weight in both active and slow-growing *E. coli* cultures (Burton, 1998).

Furthermore, recent quantitative proteomics studies have estimated that an actively growing *E. coli* cell contains approximately 28,000–36,000 ribosomes and comparable numbers of hibernation factors, including 2,000 copies of HPF and 7,900 copies of RaiA, although only 199 copies of RMF (Schmidt et al., 2016). Thus, actively growing cells seem to bear a large pool of available hibernation factors, sufficient to bind approximately a third of all cellular ribosomes (Table 2). Recent studies suggest that dedicated mechanisms exist to constrain hibernation factors in an inactive state in actively growing cells to prevent them from interfering with protein synthesis. For example, *S. aureus* produces YwlG under normal growth conditions, which acts as an inhibitor that sequesters the hibernation factor HPF (Ranava et al., 2022). Similarly, *Saccharomyces cerevisiae* has been shown to inactivate the hibernation factor Serbp1/Stm1 via phosphorylation by the TOR kinase (Shetty et al., 2023). Furthermore, mutations of ribosome-binding residues of Serbp1 that are phosphorylated by TORC1 cause a delay in the resumption of protein synthesis in nutrient-deprived cells once they are transferred to fresh media (Shetty et al., 2023). This suggests that TORC1-mediated regulation of ribosome activity might not be limited to inducing dormancy, but also play a role in the reactivation of ribosomes upon nutrient replenishment.

**Table 2:** Estimated protein copy numbers in *Escherichia coli* during active growth compared to the stationary phase.

Protein	Active growth (LB)	Stationary phase (1 day)
Protein copy number per cell		
**Ribosomes (ribosomal proteins)**		
Ribosomal protein uL1	28,189	2,255
Ribosomal protein uS2	36,238	2,432
**Translation factors**		
EF-G	31,959	1,874
EF-Tu	103,701	6,165
**Hibernation factors**		
RMF	199	3,538
RaiA	7,880	11,711
HPF	1,991	4,011
YqjD	2,203	6,209
ElaB	1,968	4,052
YgaM	756	793
Sra	640	5,851
**Other factors involved in ribosomal stress response**		
RelA	12	2
SmpB (tmRNA)	38	12
Etta (yjjK)	2,984	203
RsfA (ybeB)	2,813	480
**Factors of RNA/ribosome degradation**		
Rnase R	61	7

When cells exhaust the available nutrients and transition to stationary phase, the synthesis of ribosomes slows down and cells degrade a significant fraction of their ribosomes shortly after nutrient depletion (Adachi and Sells, 1979; Davis et al., 1986). In *E. coli*, where cells are estimated to translate an average protein in just 20 s, the transition to ribosome hibernation is known to take about 1 min. Mass spectrometry studies show that after 24 h in stationary phase, *E. coli* cells contain about 2,000 ribosomes, whereas the amount of hibernation factors HPF, RaiA and RMF increases to 4,000, 11,700, and 3,500, respectively (Schmidt et al., 2016). Thus, hibernation factors greatly outnumber ribosomes in these conditions, explaining their ability to bind essentially all cellular ribosomes during prolonged periods of metabolic inactivity. Notably, hibernation factors also substantially outnumber other factors involved in ribosome-associated stress responses. For example, stationary phase *E. coli* cells contain just 2 detectable copies of the protein RelA that is involved in cellular responses to stress or nutrient depletion (Schmidt et al., 2016), which corresponds stoichiometrically to just 0.01% of cellular ribosomes (Table 2).

## Ribosomes are not the only enzymes that may hibernate: the case of RNA polymerases

**Figure 4:**
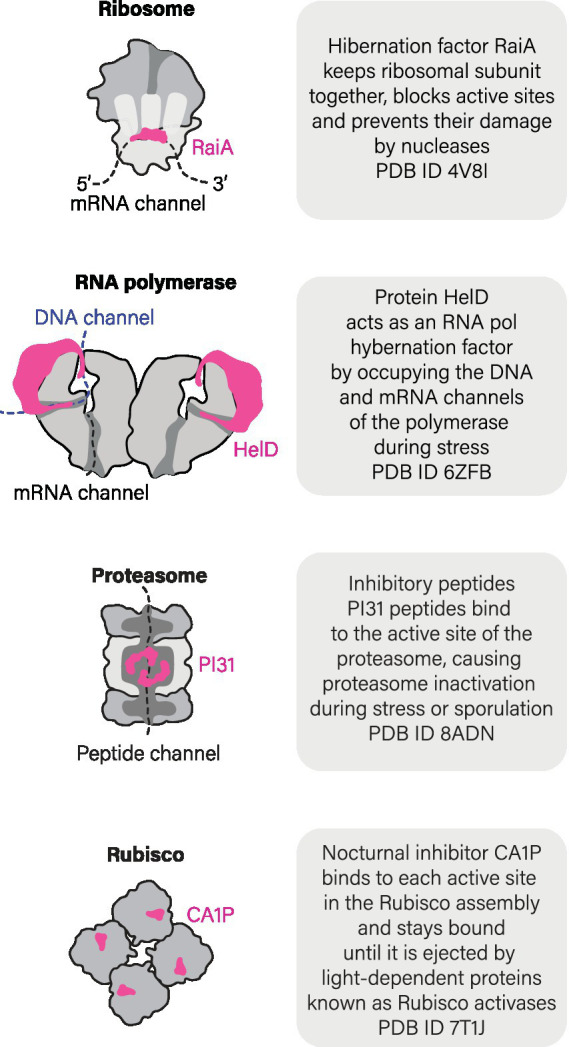
Ribosomes are not the only molecules that can hibernate.

Importantly, states of molecular hibernation are not limited to ribosomes but also extend to other essential enzymes (Figure 4). Another well-characterized case of hibernating enzymes includes RNA polymerases. In both eukaryotes (*S. cerevisiae*) and bacteria (*M. smegmatis*), RNA polymerases have been shown to hibernate by forming inactive dimers or octamers (Fernández-Tornero, 2018; Kouba et al., 2020; Pei et al., 2020; Aibara et al., 2021; Heiss et al., 2021; Morichaud et al., 2023). In yeasts, hibernation of RNA polymerase I was explored both *in vitro* and *in vivo* (Torreira et al., 2017). This hibernation is induced through the formation of dimers, where the flexible stalk of RNA polymerase I from one molecule is inserted into the DNA-binding channel of another molecule within the dimer. As a result, RNA polymerase I becomes inactive. When dormant yeast cells are brought back to an optimal environment, RNA polymerase I dimers get disassembled with the help of the protein Rrn3 that prevents the stalk from acting as a DNA tunnel-binding factor (alongside other activities of Rrn3) (Torreira et al., 2017).

Furthermore, recent studies have revealed that bacteria *M. smegmatis* and *B. subtilis* possess a dedicated hibernation factor for RNA polymerase, protein HelD (Kouba et al., 2020; Pei et al., 2020). When cells experience starvation and stress, HelD acts in a similar fashion to ribosome hibernation factors by occupying the active sites of RNA polymerase, such as DNA- and RNA-binding channels (Figure 4). Thus, at least two molecular machines of a living cell were shown to hibernate in association with dedicated and genetically encoded hibernation factor proteins.

## Small molecules can act as hibernation factors too: the case of Rubisco

A few other enzymes, such as plant catalases or Rubisco enzyme, were shown to enter a hibernation-like state in response to associating with small molecules endogenously produced by plant cells in response to cold shock or darkness (Patterson et al., 1984; Gutteridge et al., 1986). Most extensively, this process has been studied for Rubisco, the plant enzyme responsible for converting carbon dioxide into organic compounds. During night time, when the light becomes unavailable for promoting the photosynthesis, Rubisco markedly reduced its activity. In some plant species, this inhibition was shown to be caused by the intracellular accumulation of the small molecule 2-carboxy-D-arabinitol 1-phosphate (CA1P) during darkness and low light, progressively binding to the active site of Rubisco (Moore and Seemann, 1994; Liu et al., 2022; Orr et al., 2023). As light increases, CA1P is removed from Rubisco by a specialized protein called Rubisco activase, while a specific phosphatase known as CA1Pase inactivates CA1P through dephosphorylation, producing a CA molecule that cannot bind to Rubisco (Orr et al., 2023). In addition to CA1P, several other small molecules are currently being investigated as condition-specific endogenous inhibitors of plant Rubisco. These include phosphorylated sugar molecules known as XuBP (Salvucci and Crafts-Brandner, 2004), PDBP (Bracher et al., 2017), and CTBP (Parry et al., 2008). While it is unknown how many enzymes in nature undergo a similar hibernation mechanism, this example illustrates that, in addition to genetically encoded hibernation factor proteins, some enzymes can hibernate by associating with endogenously produced small molecules.

## How common is hibernation of biological molecules?

We do not know how many enzymes can hibernate. But the growing evidence suggests that molecular hibernation may be a common quality among essential enzymes to enter, withstand, and emerge from a wide variety of cellular stresses. For instance, dedicated inhibitors that are induced by stress and starvation—among other factors—have been identified not only for ribosomes and RNA polymerases, but also for the ATP synthase of eukaryotic mitochondria (protein IF1) (Pullman and Monroy, 1963; Gu et al., 2019) and proteasomes (protein PI31) (Chu-Ping et al., 1992; Jespersen et al., 2022), although their role in molecular hibernation is still a matter of debate. Nevertheless, given a growing number of instances of apparent molecular hibernation, it is very plausible that living cells employ an array of undiscovered and intricate mechanisms to prepare their most precious molecules for extended periods of inactivity—in a state of a delicate balance between being alive and dead that we call hibernation.

In the story of the Tower of Babel, God decides to punish arrogant people by inventing new languages to prevent them from understanding each other. It seems that a similar issue exists in the field of ribosome hibernation, where homologs of the same protein have different names in different organisms or even in the same organism (with *Mycobacterium smegmatis* being the prime example). To help our readers, we have created a catalog of the most common names of proteins from the HPF/RaiA family. We show that this craving for pseudonyms arises from independent discoveries of the same protein in different species or is driven by the fact that some species encode more than one isoform of HPF/RaiA factors, creating a need to discriminate between them.

## Author contributions

KH-B: Conceptualization, Writing – original draft, Writing – review & editing. LIC: Conceptualization, Writing – original draft, Writing – review & editing. SVM: Conceptualization, Writing – review & editing.
